# Just Before I Recognize Myself: The Role of Featural and Multisensory Cues Leading up to Explicit Mirror Self‐Recognition

**DOI:** 10.1111/infa.12236

**Published:** 2018-03-02

**Authors:** Maria Laura Filippetti, Manos Tsakiris

**Affiliations:** ^1^ Department of Psychology University of Essex; ^2^ Psychology Department Royal Holloway University of London; ^3^ The Warburg Institute School of Advanced Study University of London

## Abstract

Leading up to explicit mirror self‐recognition, infants rely on two crucial sources of information: the continuous integration of sensorimotor and multisensory signals, as when seeing one's movements reflected in the mirror, and the unique facial features associated with the self. While visual appearance and multisensory contingent cues may be two likely candidates of the processes that enable self‐recognition, their respective contribution remains poorly understood. In this study, 18‐month‐old infants saw side‐by‐side pictures of themselves and a peer, which were systematically and simultaneously touched on the face with a hand. While watching the stimuli, the infant's own face was touched either in synchrony or out of synchrony and their preferential looking behavior was measured. Subsequently, the infants underwent the mirror‐test task. We demonstrated that infants who were coded as nonrecognizers at the mirror test spent significantly more time looking at the picture of their own face compared to the other‐face, irrespective of whether the multisensory input was synchronous or asynchronous. Our results suggest that right before the onset of mirror self‐recognition, featural information about the self might be more relevant in the process of recognizing one's face, compared to multisensory cues.

The ability for mirror self‐recognition is viewed as a fundamental milestone in the development of self‐awareness. Normally, when encountering our mirror reflection, we recognize the facial features we see and identify them as belonging to the self. This is true despite the continuous, albeit subtle, visual changes that our face undergoes over the course of years. The increasing familiarity with our unique visual appearance is indeed a crucial type of cue for self‐identification. However, to maintain a stable representation of oneself and one's own identity, the ability to recognize oneself in a mirror also involves the integration of tactile, proprioceptive, and motor events which need to be matched with the visual information provided by the reflection (Lewis & Brooks‐Gunn, 1979; Rochat, 2003). The role of multisensory contingency and visual appearance for self‐awareness has been studied in infants (for a review, see Rochat, 2011), nonhuman primates (Gallup, 1970; Povinelli, Rulf, Landau, & Bierschwale, 1993; Povinelli et al., 1997), and adults (Platek, Thomson, & Gallup, 2004; for a review, see Tsakiris, 2016).

Developmentally, a large set of studies have indeed demonstrated infants' early abilities to discriminate visual‐proprioceptive contingency arising from their own movements (Bahrick & Watson, 1985; Morgan & Rochat, 1997; Reddy, Chisholm, Forrester, Conforti, & Maniatopoulou, 2007; Rochat & Morgan, 1995; Schmuckler & Jewell, 2007; Watson, 1994), as well as perfectly synchronous multisensory cues related to the body (Filippetti, Farroni, & Johnson, 2016; Filippetti, Johnson, Lloyd‐Fox, Dragovic, & Farroni, 2013; Zmyj, Jank, Schütz‐Bosbach, & Daum, 2011). However, while these studies show that multisensory contingency becomes functional quite early in life, the ability to discriminate this information does not necessarily imply that the infant is able to recognize these movements and body parts as belonging to the self (Bremner, Holmes, & Spence, 2012; Lewis & Brooks‐Gunn, 1979). Independently of multisensory contingency that can support momentary recognition, infants have to also build a more diachronic and permanent representation of the self, as, for example, when recognizing one's photograph in the absence of current multisensory or sensorimotor input (Lewis & Brooks‐Gunn, 1979; Rochat, 2009). For example, to recognize our own face as standing for ourselves, we must be familiar with our own facial features and be able to successfully associate them to ourselves every time we encounter our own mirror reflection (Lewis, 2011; Lewis & Brooks‐Gunn, 1979; Rochat, 2009, 2011), eventually building a more permanent offline (i.e., mnemonic) representation of our appearance (Rochat, 2003).

To investigate the ontogenesis of self‐recognition, the reference paradigm has been developed by Gallup (1970) with chimpanzees, and by Amsterdam (1972) with human infants. In Gallup's original investigation, the “mark test of mirror self‐recognition” (Gallup, 1970) entailed placing a red spot on the chimpanzee's forehead and testing whether, when faced with a mirror, the animal attempts to reach for and remove the spot. In 1972, Amsterdam developed a similar approach for testing mirror self‐recognition in human infants. In this revised task, infants were marked on the side of the nose with a rouge by their mother and subsequently exposed to the mirror (for limitations of this procedure, see Gallup, 1994). The presence of self‐directed behavior in front of a mirror is often operationalized as an understanding that the face seen in the reflection is one's own face (Rochat, 2003, 2009). Evidence suggests that from 18 to 24 months, infants show a specific set of mark‐directed (e.g., touching the mark) and self‐conscious (e.g., coy/embarrassment) behaviors (e.g., Amsterdam, 1972; Bertenthal & Fischer, 1978; Brooks‐Gunn & Lewis, 1984; Lewis & Brooks‐Gunn, 1979). This is considered a demonstration of the presence of self‐recognition, meaning that infants are not only able to recognize the contingent and featural properties of their own reflection, but they can also associate them as standing for themselves (Legerstee, 1999; Lewis & Brooks‐Gunn, 1979; Rochat, 2003).

This test has been often criticized for providing a reductionist picture of self‐conscious behavior in development and for disregarding the role of self‐exploration of the multisensory correspondences (see, e.g., Rochat, 2011). Nevertheless, the presence of a specific set of skills and behaviors when encountering a mirror around 2 years of age suggests that infants seem to acquire new crucial knowledge about the self at this developmental age. Specifically, these studies seem to suggest that the maturation of multisensory contingency precedes recognition of visual appearance (Lewis & Brooks‐Gunn, 1979), whereby infants first become familiar with this perfect multisensory matching and only later on they build a representation of their own face as standing for themselves (Bigelow, 1981). In line with this proposal, Povinelli, Landau, and Perilloux (1996) modified the classic mirror test by introducing a temporal delay of 3 min on the video footage of 2‐, 3‐, and 4‐year‐old toddlers. They found that only 4‐year‐olds show a significant percentage of reaching behavior toward the sticker, in the attempt of removing it (Povinelli et al., 1996). Miyazaki and Hiraki (2006) extended Povinelli and colleagues' findings and demonstrated that self‐recognition is facilitated when children are allowed to experience the contingent relationship between their own actions and visual feedback (Miyazaki & Hiraki, 2006). While the use of videos instead of mirror reflection might have impaired children's performance at this self‐recognition task (Suddendorf, Simcock, & Nielsen, 2007), the understanding that the current self exists beyond the perfect matching of visual‐proprioceptive correspondences seems to gradually emerge from the repetitive exposure to multisensory contingent information.

The conundrum of the interplay between contingent cues and featural information for self‐recognition becomes even more critical when we look at how manipulations of these components can produce a change in self‐identification in adults, where a robust representation of how we look like is well in place. In these studies, the simultaneous stroking of the participant's face with another person's face leads to the incorporation of a certain percentage of the other‐face into one's own self‐identification (Paladino, Mazzurega, Pavani, & Schubert, 2010; Serino et al., 2015; Sforza, Bufalari, Haggard, & Aglioti, 2010; Tajadura‐Jiménez, Grehl, & Tsakiris, 2012; Tsakiris, 2008). Studies that have explored the neural underpinnings of self‐recognition in adults have further highlighted that processes other than mere visual perception are engaged in the identification of one's own face in the mirror. For example, Apps, Tajadura‐Jiménez, Sereno, Blanke, and Tsakiris (2013) used fMRI during the enfacement illusion to demonstrate the interplay between unimodal and multisensory brain areas, such as the inferior occipital gyrus, the right temporo‐parietal junction, and the intraparietal sulcus. Serino et al. (2015) employed an ecological version of the enfacement illusion to show that after being exposed to visuo‐motor synchrony in a virtual mirror setup, online sensory–motor activation was linked to activity over inferotemporal–occipital areas of the cortex. Therefore, it seems that our mental representation of how we look like is flexible and susceptible to a number of sensory signals (i.e., motor, proprioceptive, tactile, and visual signals), suggesting that multisensory contingency is a mechanism that is not only involved in the construction of the mental representation of one's own face (see, e.g., Platek et al., 2004), but also continuously contributes to the update of our self‐representation (Tajadura‐Jiménez, Grehl, et al., 2012).

Despite the apparent crucial role that both featural information and multisensory contingent cues play in self‐recognition, the relation between these two mechanisms in the developmental phase preceding explicit mirror recognition remains poorly understood. In fact, it is unclear whether bottom‐up multisensory cues such as proprioceptive and tactile inputs are necessary for infants in this process, or whether building up gradual experience with one's own facial feature is sufficient for the emergence of self‐recognition. Studies that have specifically investigated children's ability to recognize their pictorial representation have reported controversial results. While some demonstrated that this ability comes later in development, compared to the ability to recognize oneself in front of a mirror (Courage, Edison, & Howe, 2004; Legrain, Cleeremans, & Destrebecqz, 2011; Lewis & Brooks‐Gunn, 1979), other studies have shown that 4‐ to 9‐month‐old infants prefer to look at the image of a peer compared to their own image (Bahrick, Moss, & Fadil, 1996; Legerstee, Anderson, & Schaffer, 1998; Rochat & Striano, 2002). Nielsen, Dissanayake, and Kashima (2003) explored the looking behavior of 9‐ to 24‐month‐old infants in a longitudinal study, while presented with a prerecorded video of their face and another infant's face, which were marked with red lipstick in one condition and unmarked in the other. From 12 months of age, after each looking‐time session the authors also investigated the infants' performance at the mirror test. They found that a visual preference for the self‐face only becomes apparent with the onset of mirror self‐recognition, at 18 and 24 months of age. That is, infants that show mark‐directed behavior at the mirror test also display a visual preference for their own image at that testing session, irrespective of the experimental condition (marked versus unmarked; Nielsen et al., 2003). The present experiment aimed to further tackle this issue, by investigating the contribution of facial appearance and multisensory integration in mirror self‐recognition.

We measured the looking behavior of 18‐month‐old infants, presented with previously taken pictures of themselves and a peer, which were systematically and simultaneously touched on the cheek with a hand (see Addabbo et al., 2015 for a similar experimental paradigm). While watching the video display, the infant's own cheek was touched either in synchrony or out of synchrony by a brush. Subsequently, the infants underwent the mirror‐test task. We hypothesized that infants who did not show any mark‐directed behavior at the mirror test (nonrecognizers) would show a preferential looking to the self‐face picture compared to the other‐face picture. In fact, in the process of learning to recognize the self, we expected that infants who do not pass the mirror test would pay more attention to their own facial features compared to another infant's visual appearance (Nielsen et al., 2003). We also hypothesized that this increased looking to the self would be apparent in the synchronous condition, as opposed to the asynchronous condition. If multisensory integration represents a valuable learning tool for differentiating between what pertains to the self versus the other (Tsakiris, 2016), we expected nonrecognizers to be more attuned toward the self‐face when experiencing visual–tactile synchrony, as opposed to asynchrony.

## Methods

### Participants

Infants were recruited from a database of parents who had agreed to participate in child development studies. Fifty‐one infants were recruited and invited to participate to the study. Of those, twenty‐six 18‐month‐old infants (15 girls, 11 boys, mean age = 18 months and 2 days, *SD* = ±8.93 days) completed all stages of the study and are included in the final analysis. The 28 infants who are excluded from the final analysis were excluded on the basis of the following issues: equipment failure (1), lack of behavioral data due to fussiness (4), or lack of interest on watching the video screen accompanied by increased interest toward the brush during the stroking action, which prevented us to disentangle the role of visual–tactile synchrony (23). Prior to testing, informed consent was obtained from all parents. Testing only took place if the infant was awake and alert. The local Ethics Committee (Department of Psychology, Royal Holloway University) approved the study protocol.

### Stimuli and procedure

#### Looking‐time task

Infants were tested in a sound‐attenuated room and sat on their parent's lap. The distance between the screen and the infant's head was approximately 100 cm. Stimuli were displayed on a 32″ screen monitor. Parents were asked to refrain from talking and interacting with the infant during the stimulus presentation.

**Figure 1:**
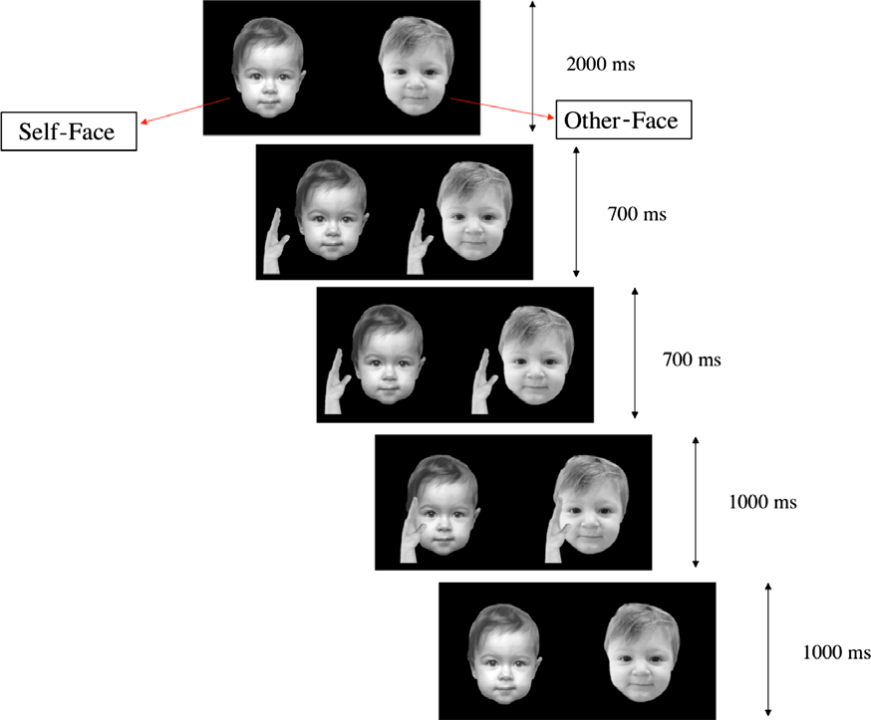
Illustrative example of the experimental paradigm used for the preferential looking‐time task. The two hands appeared on the screen after 2 sec of picture presentation. The “touch” action lasted approximately 2.5 sec (approaching action: 700 msec followed by 700 msec; stroking: 1 sec). One further second of stimulus presentation showed both pictures without hands. Each experimental condition comprised five displayed touches to both faces, and the two trials were 30 sec long.

The design comprised two experimental conditions and one baseline condition. The experimental stimuli consisted of previously taken pictures of the infant tested and a peer that were stroked on their left cheek by a hand every 6 sec by a virtual hand. Using a preferential looking procedure, we displayed both photograph pictures side by side (self‐face and other‐face). Pictures were taken against a black background, asking the parent to sit the baby on a chair. The two hands appeared on the screen after 2 sec from stimulus presentation. The entire “touch” action lasted approximately 2.5 sec (“hands approaching” action: 700 msec followed by 700 msec; “stroking” action: 1 sec), followed by 1 further second of stimulus presentation where the hands were absent (Figure 1). Each experimental condition comprised five displayed touches to both faces, and the two trials were 30 sec long. The other‐infant face was selected using a yoked‐controlled design; that is, the photograph of each infant is used as the “peer stimulus” for the next infant (Bahrick & Watson, 1985; Legerstee et al., 1998). This method allows balancing for different visual features perceived by the infants in each condition (Legerstee et al., 1998; Nielsen et al., 2003). During both experimental conditions, the experimenter, hidden behind the infant, used a soft paintbrush to touch the infant face on her right cheek, either in synchrony or in asynchrony with the stroke seen on the video screen. Therefore, the two experimental conditions differed only in that one was time delayed relative to the seen touch by 3 sec. In other words, while in the synchronous condition the experimenter applied the touch on the cheek simultaneously with the touch seen on the video, in the asynchronous condition the same brush event occurred with a 3‐sec lag from the touch occurring on the screen (Gergely & Watson, 1999; Zmyj, Hauf, & Striano, 2009). To ensure infants' attention was maintained throughout the experimental session, a 5‐sec baseline (full color, static images of animals and objects) appeared at the beginning of the experimental session and after 30 sec of the paired picture presentation (between the two experimental conditions). The position of the pictures (right or left sides of the screen) and the order of the two conditions were counterbalanced between infants. The experimental task lasted about 3 min and was run using E‐Prime 2.0.10 software. Stroking of the infant's cheek was manually delivered by the experimenter using a soft medium‐sized paintbrush (width = 25 mm) and was always delivered in the specularly congruent location. Each stroke lasted approximately 1 sec. As infants were seen to lose their attention during the preferential‐looking task, we decided to conduct this part of the study first, to enhance the chances of data collection (see also Nielsen, Suddendorf, and Slaughter, 2006 for similar experimental procedure).

#### Mirror‐test task

After this experimental session, the experimenter prepared the setting for the mirror‐test task (Amsterdam, 1972). The caregiver was asked to sit on a chair away from the infant's sight and from a large mirror (to avoid that their reflection could interfere with the task). A video camera was directed at the mirror from an angle of the room. The mirror‐test task comprised a warm‐up phase and a testing phase. To familiarize with the mirror, in the warm‐up phase the experimenter encouraged the infant to approach the mirror. This phase was concluded once the infant made eye contact with her reflection twice and at least once for 2 sec (Asendorpf & Baudonnière, 1993; Kristen‐Antonow, Sodian, Perst, & Licata, 2015). Based on this exclusion criterion, we excluded from the final sample two infants (two boys), as they could not be brought to focus on their mirror reflection (Asendorpf & Baudonnière, 1993; Asendorpf, Warkentin, & Baudonnière, 1996; Kristen‐Antonow et al., 2015). Subsequently, a free play period allowed the experiment to discreetly apply a spot of odorless, nontoxic, water‐soluble, red face paint on the cheek of the infant, in preparation for the testing phase of the mirror task. We chose to apply the mark on the cheek to keep the looking‐time task and the mirror‐test task as consistent as possible (as in the looking‐time task infants watched and experienced touch on that region of the face). No infant reached for the mark prior to the introduction to the mirror, indicating that they did not feel the marking event. After approximately 5 min, the experimenter encouraged again the infant to approach the mirror (testing phase). Infants who spontaneously displayed at least one of the marker behaviors listed in Table 1 were considered to pass the test.

**Table 1:** List of Marker Behaviors, Used to Operationalize the Mirror Test in the Present Experiment

Nonverbal behavior
Touch mark/nose
Touch the region of the mark
Try to touch mark (including opposite cheek)
Staring at the mark, accompanied by self‐conscious behavior (e.g., shame, coyness)

### Data analysis

Based on the video recordings, an observer (blind to the conditions) coded how long each infant looked at each of the two side‐by‐side pictures. For our dependent measures, we computed the mean of total looking time spent orienting toward the self‐face picture and the other‐face picture, in both synchronous and asynchronous conditions. Inter‐rater reliability analysis was performed by a second observer blind to both the experimental conditions and the hypotheses on 20% of the sample and revealed a score of Pearson's *r *=* *0.80. For the mirror‐test task, one observer coded the behavior offline from the recorded observations. Recognition was scored as either present or absent, based on the marker behaviors listed in Table 1. The final sample comprised 26 infants: 15 (12 girls, three boys) recognizers and 11 (four girls, seven boys) nonrecognizers. Verbalizations were not considered a marker behavior of self‐recognition in the present experiment, as the same verbalizations occurred in both familiarization and testing phases—for example, saying “Baby” (with the exception of one infant, who in the testing phase touched the mark and said “Face”). Inter‐rater reliability analysis was performed by a second observer blind to the hypotheses on 20% of the sample, and the observed agreement between the two coders was 100%.

## Results

We first tested with a repeated‐measures ANOVA whether the side of the screen, the order of condition, the gender of the infant, or the gender of the peer in the looking‐time task had a significant effect. None of these factors revealed any significant effects, and therefore, data were averaged across these. We next performed a repeated‐measures ANOVA with Stroking mode (Synchronous verus Asynchronous) and Face Identity (Self verus Other) as within‐subject variables and Self‐recognition Group (recognizers verus nonrecognizers) as between‐subject factor. The main effects of Stroking mode, *F*(1, 24) = 0.02, *p *=* *.89, Face Identity, *F*(1, 24) = 1.39, *p *=* *.25, and Self‐recognition Group, *F*(1, 24) = 0.05, *p *=* *.82, were not significant.

**Figure 2:**
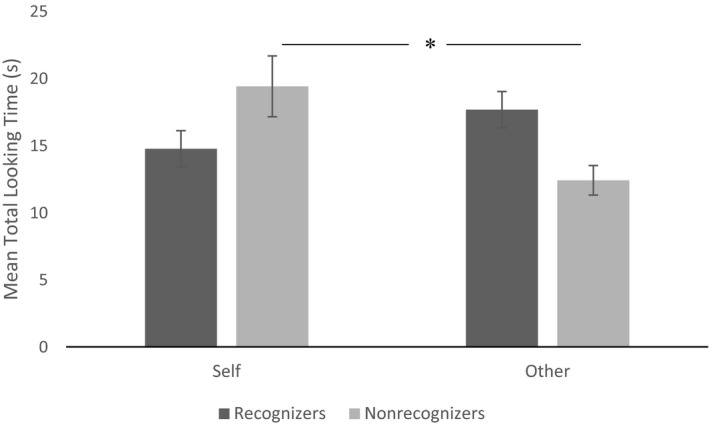
Mean total looking time to the self and to the other of infants who passed (recognizers, in dark gray) and did not pass (nonrecognizers, in light gray) the mirror‐test task. Only nonrecognizers looked significantly longer at the self‐face compared to the other‐face. Error bars show standard errors (*SE*).

The three‐way interaction Self‐recognition Group × Stroking mode × Face Identity was not significant, *F*(1, 24) = 0.06, *p *=* *.80, indicating that the type of multisensory stimulation (i.e., synchronous or asynchronous) when watching self‐face or other‐face did not influence recognizers and nonrecognizers differently. However, we found a significant interaction, Face Identity × Self‐recognition Group, *F*(1, 24) = 8.14, *p *=* *.009, *η* ^*2*^ = 0.25. Using planned pairwise comparisons to interpret the interaction, we found that while infants that passed the mirror test did not show any preferential looking to the self‐ or other‐face across the two conditions, *t*(14) = −1.34, *p *=* *.20, infants that did not pass the test looked significantly more to the self‐face, compared to the other‐face, *t*(10) = 2.53, *p *=* *.03, *d *=* *1.18 (Figure 2). Nonparametric analysis (Wilcoxon signed rank test) on the number of nonrecognizer infants who showed a tendency to look longer at the self‐face image (*N* = 10) showed that the median looking‐time rank to the self‐face was statistically significantly higher than the median to the other‐face, *Z* = −2.80, *p* = .005. Nonparametric statistics (Wilcoxon signed rank test) on the number of recognizer infants who showed a tendency to look longer at the other‐image (*N* = 9) indicated that the median looking‐time rank to the other‐face was significantly higher than the median to the self‐face, *Z* = 2.66, *p* = .008.

Additionally, we investigated the distribution of first look and longest look duration in the sample. We found no significant differences between the first look to the self or to the other in recognizers and nonrecognizers, *χ* ^*2*^(1, *N* = 26) = 0.735, *p* = .391—recognizers: self (26.9%), other (30.8%); nonrecognizers: self (26.9%), other (15.4%). Similarly, we found no significant differences between the longest look to the self or to the other in recognizers and nonrecognizers, *χ* ^*2*^(1, *N* = 26) = 1.766, *p* = .184—recognizers: self (26.9%), other (30.8%); nonrecognizers: self (30.8%), other (11.5%) (see Table 2 for number of infants showing the phenomena).

**Table 2:** Distribution of the First Look and Longest Look in Recognizers and Nonrecognizers

	First look		Longest look	
	Other‐face	Self‐face	Other‐face	Self‐face
Recognizers	8	7	8	7
Nonrecognizers	4	7	3	8

Nonrecognizers' first look and longest look were to the self‐face.

## Discussion

The ability for self‐face recognition is a key aspect of self‐awareness and identity (Gallup, 1970; Rochat, 2011). To recognize our face as distinct from others, we must associate the motor, proprioceptive, and tactile input we experience with its mirrored compatible visual event, and gradually become familiar with the featural information uniquely associated with our face. While previous developmental work has independently investigated the respective role of visual appearance and multisensory contingent cues for self‐awareness, little is known about their concurrent interplay in the gradual acquisition of self‐recognition.

In the current study, 18‐month‐old infants watched their face and another infant face being touched by a hand either in synchrony or in asynchrony with a touch experienced on their own cheek. Subsequently, the infants underwent the mirror‐test task, and their reaction in front of a mirror to a mark applied on their cheek was measured. We hypothesized that infants who did not show any mark‐directed behavior during the mirror test would also display a visual preference for their own face and that this preference would be more apparent in the visuo‐tactile synchronous, compared to the asynchronous, condition. In line with our first hypothesis, we found that infants who did not show any mark‐directed behavior at the mirror test (i.e., infants that were coded as nonrecognizers) spent significantly more time looking at the picture of their own face compared to the other‐face. However, this visual preference was present irrespective of whether the visual stroking action seen on the screen was synchronous or asynchronous to the touch they felt on their own face. Based on these findings, we demonstrate that in the absence of explicit mirror self‐recognition, infants pay more attention to their own familiar facial features compared to unfamiliar facial features of others. In contrast, multisensory contingent cues (here expressed as synchronous visuo‐tactile signals) may not be the primary or the only signals used to discriminate between self and other facial features at this specific developmental stage. However, given the limited sample size, it is important that future research replicate the present findings.

Extensive work on the role of multisensory contingent cues for self‐awareness comes from both the infant and adult literature, suggesting the fundamental role of this information for the development of our bodily self (e.g., Bahrick & Watson, 1985; Filippetti et al., 2013; Watson, 1994; Zmyj et al., 2009, 2011), as well as the update of body ownership (e.g., Blanke & Arzy, 2005; Botvinick & Cohen, 1998; Tsakiris & Haggard, 2005) and self‐identification (e.g., Tajadura‐Jiménez, Grehl, et al., 2012; Tajadura‐Jiménez, Longo, Coleman, & Tsakiris, 2012; Tsakiris, 2008). However, while adult studies can provide us with evidence of individuals' ability to associate that specific body part as belonging to the self (through report and questionnaires), infants' studies are obviously limited by their inability to report their experience. Hence, the infant's ability to show a visual preference to contingent versus noncontingent multisensory cues may not be linked to the ability to refer to her own body when detecting the matching between the two sensory modalities (Bremner et al., 2012). As Lewis and Brooks‐Gunn (1979) suggested, while self‐recognition might at first be highly dependent on both visual appearance and visual‐proprioceptive contingency, from 15 to 18 months infants might start relying more on their own facial features to recognize their mirror reflection as standing for themselves (Lewis & Brooks‐Gunn, 1979). Thus, the absence of a preference for synchronous visuo‐tactile cues in our study might be explained by this developmental shift in attention. However, as the present study used visuo‐tactile information in the absence of any motor signal (e.g., visual and proprioceptive inputs), it is possible that the rather unfamiliar experience of being touched on the cheek by a brush might have driven attention away from the synchronicity of the sensory information. To rule out this possibility, further studies should overcome the technical limitation of using visuo‐proprioceptive and/or sensorimotor synchronous cues between self and other and develop experimental paradigms that allow for the use of the ecological and more familiar sensorimotor contingency. The use of virtual reality (VR) experiments could potentially provide a crucial insight into the underlying mechanisms of self‐recognition, through the manipulation of synchronous and asynchronous visuo‐motor contingencies applied to the self and other. As shown by Serino et al. (2015) with adults, the use of virtual mirror setups could disentangle the respective contribution of visual, tactile, and proprioceptive signals in the development of mirror self‐recognition, as well as the distinction between self and others.

In the present study, we compared performance in the mirror test with looking behavior in response to pictorial representations of the self and the other. Previous studies investigating children's ability to recognize their pictorial representation have reported controversial results, with some research suggesting early self‐other discrimination abilities (Bahrick et al., 1996; Legerstee et al., 1998; Rochat & Striano, 2002) and other demonstrating later development (Courage et al., 2004; Legrain et al., 2011; Lewis & Brooks‐Gunn, 1979). In a longitudinal study, Nielsen et al. (2003) showed that a visual preference for the self‐image only becomes apparent with the onset of mirror self‐recognition. In our experiment, we found that infants who did not pass the mirror test looked significantly more to their own face, compared to the peer's face, perhaps suggesting the presence of an increased interest for one's own facial features, coincident with the gradual emergence of the ability to recognize themselves in front of a mirror (Courage et al., 2004).

It should be noted that the task demands of a preferential looking‐time paradigm (such as the one we used here) are not the same as the explicit mirror self‐recognition task. Preferring to look at what is familiar is different from touching or removing the mark as a sign of self‐awareness. The latter may reflect an active engagement with one's appearance to modify it. The former task (i.e., preferential looking) was designed to evaluate the respective contributions that multisensory input and visual familiarity are making on infant's viewing preferences. Given these differences across the tasks, we set out to investigate whether it was visual familiarity or multisensory contingency that would be associated with performance in explicit self‐recognition. Our results suggest that infants who do not pass the explicit self‐recognition task also prefer to look at their own face: This preference may reflect the fact that nonrecognizers have not yet established a robust enough representation of their own visual appearance and are using such instances, independently of the pattern of multisensory stimulation as our results show, to further consolidate the mental representation of their own face. Once this representation is robust enough, infants would no longer show a visual preference for their own face within the social context of our first task, and this behavior would also be associated with explicit self‐recognition.

Overall, our results contribute to the growing literature that investigates the link between familiarity to one's own facial features and the ability to exhibit explicit mirror self‐recognition. However, assuming that the mirror‐test is a valid measure of self‐recognition (for critics on the validity and meaning of the test, see Bahrick et al., 1996; Broesch, Callaghan, Henrich, Murphy, & Rochat, 2011; Heyes, 1994, 1995; Rochat, 2007, 2009), we suggest that the way infants process their own self‐image changes during the first 2 years of life. In line with Nielsen et al. (2006), evidence for a visual preference for other‐face in young infants, rather than their own face (Bahrick et al., 1996; Legerstee et al., 1998; Rochat & Striano, 2002), could be interpreted as an early discrimination between self and other on the basis of familiar visual features, perhaps a first stepping‐stone into the ability to distinguish between self‐face and other‐face beyond the multisensory input, a process that can eventually lead to a more diachronic representation of one's appearance (Legrain et al., 2011). In fact, while visuo‐motor and tactile contingency might be the strongest cue for self‐other discrimination at early stages, the presence of a visual preference for the self‐image found in our 18‐month‐old infants (as well as in Nielsen et al., 2003) might underpin a different developmental ability, namely the ability to compare the stored mental image of the self with the mirror reflection, and to eventually use efficiently a mnemonic representation of one's appearance across different contexts (e.g., recognizing one's face in photographs, videos, etc.; see Legrain et al., 2011). However, as these studies used different experimental paradigms and age ranges, it is difficult to draw any definitive conclusions about how developmental differences in preferential looking to self‐face verus other‐face might inform into this ability.

An intriguing finding of our investigation is the absence of any self‐specific visual preference shown by infants that passed the mirror test (recognizers). While these infants show a tendency to look at the other infant face, this increase in looking time is not significantly different from the looking time to the self‐face. It is perhaps noteworthy that our looking‐time task has a social context as two facial identities are presented at the same time. As our results suggest, the subgroup of recognizers have a more robust mental representation of their visual appearance and this may make their social attention to self and others more flexible, allowing them to efficiently switch and alternate their attention from the self to the other. The fact that explicit mirror self‐recognition seems to occur at the same time as the expression of social emotions (e.g., pride and shame; see Lewis, Sullivan, Stanger, & Weiss, 1989; Rochat, 2009) suggests that infants who have mastered a robust enough representation of themselves can more easily switch between being attending to others and attending to themselves. Therefore, in terms of our looking‐time variable, this ability would result in comparable looking times to self and other. The current findings have important implications for the understanding of the development of self‐recognition. Crucially, our results suggest that 18‐month‐old infants, presumably right before the onset of mirror self‐recognition, show a visual preference for their own face, compared to another peer face. As this visual preference persists irrespective of multisensory contingent cues, these findings suggest that at this age, featural information might be more relevant in the process of recognizing one's face, compared to multisensory cues. Future research should investigate this topic further by exploring how different degrees of contingency (e.g., visual, proprioceptive, and motor signals) relate to the development of self‐recognition, and how synchronous versus asynchronous multisensory stimulation applied to the self and the other contributes to the development of self‐other differentiation, as well as the implementation of social interactions.
